# Differential Somatic Cell Count as a Marker for Changes of Milk Composition in Cows with Very Low Somatic Cell Count

**DOI:** 10.3390/ani10040604

**Published:** 2020-04-01

**Authors:** Alfonso Zecconi, Francesca Dell’Orco, Diego Vairani, Nicoletta Rizzi, Micaela Cipolla, Lucio Zanini

**Affiliations:** 1 Department of Biomedical, Surgical and Dental Sciences, University of Milano, Via Pascal 36, 20133 Milano, Italy; 2 Associazione Regionale Allevatori Lombardia, Via Kennedy 30, 26013 Crema, Italy; francesca.dellorco.89@gmail.com (F.D.); d.vairani@aral.lom.it (D.V.); n.rizzi@aral.lom.it (N.R.); m.cipolla.cmh@gmail.com (M.C.); luciozanini@gmail.com (L.Z.)

**Keywords:** differential cell count, somatic cell, intramammary infection, milk composition

## Abstract

**Simple Summary:**

Recently, the availability of a high-throughput milk analyzer performing a partial differential somatic cell count (DSCC) opened new opportunities in investigations on bovine udder health. The information supplied by this new tool would be of importance in cows with a very low somatic cell count (SCC ≤ 50,000 cells/mL). Our investigation confirmed that the repeatability of the measurement allows its use under field conditions. The observational data did not find an association between DSCC and intramammary infections in very low SCC cows. However, our data showed that all the major milk components decreased as the DSCC was raised. These findings confirmed that DSCC could be a new informative tool for dairy farmers to monitor the changes in milk quality. DSCC may be suggested as a marker to identify early changes in milk composition, as a result of an alteration in milk secretion mechanisms.

**Abstract:**

The recent availability of a high-throughput milk analyzer performing a partial differential somatic cell count (DSCC) opened new opportunities in investigations on bovine udder health. This analyzer has a potential limitation on the accuracy of measurements when the somatic cell count (SCC) is below 50,000 cells/mL, values characterizing a good proportion of lactating cows in many herds. We obtained data for cows below this threshold, assessed the repeatability of these measurements and investigated the relationship between DSCC and udder health, milk composition and yield. Overall, 3022 cow milk test records performed on a Fossomatic™ 7/DC (Foss A/S, Hillerød, Denmark) were considered; 901 of them had an SCC ≤ 50,000 cells/mL. These latter samples were analyzed by qPCR to identify the presence of bacteria. Overall, 20.75% of the samples (187) were positive. However, the health status did not have any significant association with DSCC. The analysis of the association of DSCC on milk fat, protein and casein showed a significant decrease in their proportions as the DSCC increased, whereas it was not observed for milk yield and lactose. Therefore, DSCC in very low SCC cows may be suggested as a marker to identify early changes in milk composition.

## 1. Introduction

The current most simple, practical and sustainable method to monitor udder health in dairy herds is represented by individual somatic cell count (SCC), even if it does not have the same accuracy as microbiological analysis [1,2]; SCC can only suggest the presence of inflammation, but not the presence of a pathogen. Usually, under field conditions, a level of 200,000 cells/mL is considered the threshold to identify subclinical mastitis [3,4,5,6]. However, the progressive decrease in mean SCC in dairy herds worldwide has resulted in a reduction in the use of SCC to identify diseased cows. There is a consensus on the association between an increase in SCC in milk with a change in the proportion of inflammatory cells in the cell population, and it has been suggested that the amount of polymorphonuclear neutrophils (PMN) may be a more useful indicator in the evaluation of udder health than SCC [7,8,9,10,11]. Indeed, milk composition changes were observed even below 100,000 cells/mL [9,12,13,14].

This evidence supported the research aiming to apply differential somatic cell count (DSCC) as a tool to identify mastitis in combination with SCC or alone. However, one of the major obstacles to the application of DSCC in practice was the unavailability of high-throughput milk analyzers with an acceptable cost for the analysis. Microscopy is the conventional method to perform DSCC, but it is time consuming and it has poor repeatability, while flow cytometry is more efficient, but the cost of the analysis and its accuracy are critical points that prevent its application outside the research field [15].

The recent availability of a high-throughput milk analyzer (Fossomatic™ 7DC, Foss A/S, Hillerød, Denmark), able to perform a partial DSCC [16] and fully integrated with the instruments currently used to analyze individual milk samples, opened new opportunities in bovine mammary gland investigations. This analyzer is now used in several countries, but it has a potential limitation related to the accuracy of measurements when the SCC is below 50,000 cells/mL [16]. Indeed, DSCC values for samples below 50,000 cells/mL are not usually reported by the instrument, even if they are recorded. 

Our recent investigation in the Lombardy region (Italy) [17] on nearly 46,000 milk test records showed that around 25% of primiparous cows and 19% of all cows has a lactational SCC average below 50,000 cells/mL. Therefore, if DSCC data are not available for the farmer, a significant proportion of information on herd health status is missing, thus impairing the application of routine tests of this technique and the potential improvement provided by the new technology.

Thanks to the cooperation with the producer of the instrument (Foss A/S, Hillerød, Denmark), we were able to retrieve data from the analyzer for cows with an SCC ≤ 50,000 cells/mL and to develop a study under field conditions with the aim to assess the relationship between DSCC and udder health status, milk composition and milk yield in these cows. We hypothesized the that the use of DSCC is a marker for early detection of intramammary infections (IMI) and for changes in milk secretion. To support this aim, we also explored the repeatability of a DSCC in samples with a SCC ≤ 50,000 cells/mL, as well as the characteristics of the sampled cows classified according to the SCC threshold of 50,000 cells/mL. 

## 2. Materials and Methods 

### 2.1. Herd and Cow Selection

This study considered 3022 cows from 24 randomly selected dairy herds in the Lombardy region (Italy) among the 150 herds that at the time of the study already applied for DSCC testing. The sample size was estimated based on the proportion of cows with a SCC ≤ 50,000 cells/mL (20%) in Lombardy as reported in a recent study [17]. More than 95% of the cows included in the study were Italian Holstein Frisian; most of the remaining ones were Italian Brown Swiss while other breeds or cross breeds represented less than 1% of the sample. Milk samples to determine their composition and the presence of bacteria by real-time PCR (qPCR) assay were collected from September to December 2018. All lactating cows in each herd were sampled only once for this purpose. 

### 2.2. Sample Collection

To reduce the risk of contamination of the milk samples in individual cow testing, teats were carefully cleaned as required for milking before applying clusters and milk meters [18]. Individual cow sampling was performed by certified methods currently applied by the Italian Breeders Association at the laboratories of the Regional Breeders Association of Lombardy (ARAL, Italy) by means of a Lactocorder™ (WMB AG, Balgach, Switzerland). Samples were delivered refrigerated to ARAL labs the same day and divided into two aliquots: one for the milk composition analysis and the other for the qPCR assay, kept refrigerated until the analyses were performed (within 30 h from the sampling). 

### 2.3. Milk Composition Analysis

Milk analyses included protein, fat, lactose and casein content, as well as the SCC and DSCC, and were carried out on a Fossomatic™ 7DC (Foss A/S, Hillerød, Denmark). The analysis of DSCC is based on the Foss DSCC Method Cell Staining (international patent PCT/EP2010/065615-Holm, 2012) as described by Damm et al. [16]. The method allows to identify within a milk sample the macrophages (MAC) and the combination of polymorphonuclear neutrophils (PMN) and lymphocytes (LYM). Diagnostic characteristics and performances were described by Damm et al. [16]. DSCC is expressed as the combined proportion (%) of PMN and lymphocytes on the overall count of milk cells.

### 2.4. Repeatibility of the SCC and DSCC Measures

To assess the repeatability of the SCC and DSCC measures in milk test samples of ≤ 50,000 cells/mL, the coefficient of variation (CV%) was estimated by testing 5 different samples having SCC ≤ 50,000 cells/mL. The 5 samples were analyzed 20 times each on two identical Fossomatic™ 7DC instruments (Foss A/S, Hillerød, Denmark) for a total of 10 repeated measures.

### 2.5. Molecular Analysis

All samples with an SCC ≤ 50,000 cells/mL were considered for qPCR assay. Individual milk samples were analyzed using Mastit M4BDF kit (DNA Diagnostic A/S, Risskov, Denmark), following the producer’s instructions (https://dna-diagnostic.com/files/Downloads/Mastit4/Instruction_protocol_M4BDF_2017.11..01.pdf). This kit allows bacterial DNA extraction, identification and quantification of *Staphylococcus aureus*, *Streptococcus agalactiae*, *Streptococcus dysgalactiae*, *Streptococcus uberis*, *Mycoplasma bovis*, *Mycoplasma* spp., coagulase-negative staphylococci, *Prototheca* spp., *Escherichia coli*, *Klebsiella species*, *Enterococcus* and *Lactococcus lactis* using qPCR. The qPCR reactions were performed on a Stratagene Mx3005P (Agilent Technologies Inc., Santa Clara, CA, USA). To avoid the potential overestimation of positive samples due to carry-over effects during sampling, when the same bacteria species was detected in two consecutive milk samples taken from the same milking slot, the second one was considered as uninfected as suggested by Mahmmod et al. [18].

### 2.6. Cow and Milk Test Record Data

Milk and cow data were recorded by ARAL and included herdID, cowID, number of lactations (n), days in milk (d), milk yield (kg/d), SCC (cells/mL), DSCC (%) and grams per 100 g of milk (%) of protein, fat, lactose and casein. The SCC was log10-transformed to a somatic cell score (SCS, units). Cow, milk and molecular analysis data were combined in a database and statistical analyses were performed.

### 2.7. Statistical Analysis

Pearson’s correlation tests, χ2 tests and Armitage–Cochrane trend tests were applied to compare the distributions of SCC and DSCC across the whole study population by means of XLSTAT 19.4.1 software (Addinsoft, New York, NY, USA). Agglomerative hierarchical clustering, applying Euclidean distances and the unweighted pair-group average as the agglomeration method, was used to classify samples with ≤50,000 cells/mL based on their composition, and an ANOVA LSD test was applied for comparison of the means among clusters, both performed in XLSTAT 19.4.1 software (Addinsoft, New York, NY, USA). Data from cows with ≤50,000 cells/mL were also analyzed by generalized linear models applying the GLM procedure in SAS 9.4 (SAS Institute Inc., Cary, NC, USA), to identify the factors associated with the different milk traits considered. 

The models were:

(a) DSCC as a marker of IMI:

Yijk = µ + Hi + Dj + Pk + eijk;(1)

where Y = DSCC; µ = general mean; Hi = effect of IMI (i = negative, major pathogens, coagulase negative Staphylococci—CNS); Dj = effect of days in milk (DIM) (j = 5–60; 61–120; 121–180; 181–240; >240 d); Pk = effect of parity (k= 1; 2; 3; 4; >4); and eijk = residual error.

(b) DSCC, milk composition and yield:

Ymijkw = μ + Hm + Fi + Dj + Pk + Iw + eijkmw;(2)

where Y = dependent variables (SCS, milk yield, fat, protein, casein, lactose); µ = general mean; H_m_ is the random effect of the mth herd (m = 1 to 24); Fi = effect of DSCC (i = 12.2–41.2; 41.3–51.7; 51.8–60.6; 60.7–91.6%); Dj = effect of DIM (j = 5–60; 61–120; 121–180; 181–240; >240 DIM), Pk = effect of parity (k= 1; 2; 3; 4; >4), Iw = effect of IMI (i = negative, major pathogens, CNS); and eijkw = residual error.

## 3. Results

### 3.1. Repeatability

The first step of our study aimed to estimate the CV% of the SCC and DSCC in samples with ≤50,000 cells/mL, analyzing 5 different samples below that threshold on two identical instruments (Fossomatic™ 7DC, Foss A/S, Hillerød, Denmark). The results are reported in Table 1. The data shows that the CV% ranged between 3% and 7% for DSCC, values that are close to the ones calculated for SCC in the same samples. Both the DSCC and SCC CV% observed were very close to the reference values reported by the producer (<6% for samples in the range 100,000–299,000 cells/mL). 

### 3.2. Field Trial—Data Description

Overall, 3022 milk samples were collected from 24 dairy herds. Table 2, Table 3 and Table 4 summarize the main characteristics of the samples. The results showed that about one third of the samples (901; 29.8%) had an SCC ≤ 50,000 cells/mL. The mean DSCC was higher in samples with an SCC > 50,000 cells/mL when compared to samples with ≤50,000 cells/mL, being respectively 68% and 51%. The frequency of cows with ≤50,000 cells/mL (Table 3) showed a statistically negative trend as the number of lactations increases (Cochran–Armitage trend test; *p* < 0.0001). Indeed, nearly 40% of the cows in first lactation have ≤50,000 cells/mL, whereas only 17% of the cows with >4 parturitions were below this threshold. Analogously, when data were analyzed by DIM, we observed a significant decrease of cows with ≤50,000 cells/mL when DIM are >60 d (*p* < 0.0001, as per the Cochran–Armitage trend test). 

The relationship between the SCS and DSCC in the two subsamples (≤50,000 cells/mL and >50,000 cells/mL) was also assessed by Pearson’s correlation analysis. The results showed that the correlation was 0.16 (*p* < 0.05) when the ≤50,000 cells/mL data were considered. When samples with >50,000 cells/mL were considered, the correlation coefficient was 0.52 (*p* < 0.05).

### 3.3. Intramammary Infections and DSCC

**Figure 1:**
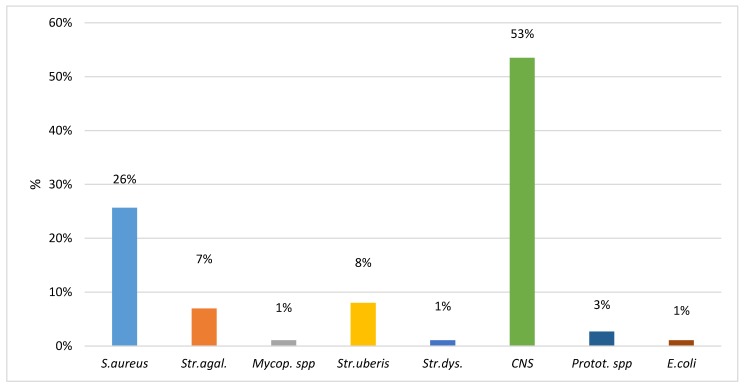
Proportion of bacteria species recovered in 187 qPCR positive milk samples with ≤50,000 cells/mL.

The samples with ≤50,000 cells/mL were analyzed by qPCR to identify the presence of bacteria. Overall, 20.75% of the samples (187) were positive and the distribution of the results is reported in Figure 1. Data show that one third of the positive samples harbored contagious pathogens, while about half of them were positive for coagulase-negative staphylococci (CNS). 

The relationship between IMI and DSCC in samples ≤50,000 cells/mL were analyzed by a simple GLM model, including health status (negative, CNS and major pathogens), parturition and DIM. The model has a low R^2^ (0.05) even if significant (*p* < 0.0001), and the health status did not show any significant effects on DSCC variability. Indeed, mean values were the following: healthy cows 49.61% ± 0.80%, CNS 48.95% ± 1.46% and major pathogens 48.50% ± 1.47%.

These results were also confirmed by the analysis of the contingency table (Table 5) comparing health status and DSCC classified by quartiles. The distribution of the samples was nearly uniform (statistically not significant following a χ2 test, α = 0.05) among the four quartiles, respectively, for the negative, major pathogen and CNS-positive samples.

### 3.4. DSCC, Milk Composition and Yield

The relationships between DSCC and milk composition, SCS and yield were analyzed by a general linear model (GLM), also including herd as random factor, while parity, days in milk and IMI were fixed factors (Table 6). The model was statistically significant for all the traits considered, with an R^2^ in the range between 55% (milk yield) and 15.4% (SCS). Herd factor was confirmed to have a significant effect on the variance of these traits and its contribution to the R^2^ was in the range 5.3%–22.9%. IMI did not show any statistical influence on the variance of the traits considered. Protein, casein and SCS were the traits where herd had the lowest influence on their variance (≤6%), while for milk yield it has the strongest effect (22.9%). DSCC values were classified in quartiles, based on the observed distribution of the values. The influence of DSCC on the variation in milk components was always highly significant (*p* < 0.0001), whereas it was not significant for milk yield and lactose. Despite our dataset included only milk samples ≤50,000 cells/mL, the statistical analysis confirmed a significant positive association between DSCC and SCS. Indeed, samples in the lowest DSCC classes also had the lowest SCS mean, whereas the highest DSCC classes showed the highest SCS mean.

The analysis of the influence of DSCC on milk fat, protein and casein showed the same pattern with a significant decrease in the proportion of the three components as DSCC increased. The decrease in the fat proportion was the most evident with an absolute decrease of 0.29% when the samples with a DSCC in the range of 12.2%–41.2% were compared to samples with DSCC in the range of 60.7%–91.6%. Protein and casein percentages showed a significant decrease when the DSCC was >51.8%, with an absolute difference between the lowest and highest DSCC class of 0.12% and 0.10%, respectively.

To confirm these relationships, data were analyzed by agglomerative hierarchical clustering, resulting in the definition of three characterized clusters, as reported in Table 7. The statistical analysis showed that Cluster A is characterized by statistically significant, lower means for DSCC, SCS and lactose, and higher means for fat, protein and casein compared with clusters B and C. Cluster C, on the other end, is characterized by statistically significant higher means for DSCC, but the opposite for fat, protein and casein when compared with the other clusters.

## 4. Discussion

### 4.1. Repeatibility

Previous studies reported a coefficient of variation (CV%) for a DSCC of 15% when the SCC was in the range of 8000–7,000,000 cells/mL [16]. The producers of the instrument considered this value too high, not recommending using it. Indeed, the relatively low number of cells in the volume of the sample (50 μL) counted by the instrument could potentially lead to higher variability when compared to samples with a higher SCC. Therefore, data on the repeatability of DSCC measures in milk samples with ≤50,000 cell/mL are not usually reported, and they are not available in the scientific literature. We observed that the proportion of cows with these SCC values were relatively high in Lombardy herds (≈20%), and the missing DSCC values decrease the interest in the application of DSCC in practice. Therefore, we were interested in assessing its repeatability because, if satisfactory, it would allow the routine measurement of DSCC in milk samples. The analytical performance observed in this study was very close to reference values and supports the use of a DSCC, also in milk samples with ≤50,000 cells/mL. Indeed, the repeatability observed was very similar for both the SCC and DSCC. Rationally, any criticism on the correctness of DSCC based on CV% in very low SCC cows should be also applied to SCC, thus invalidating a large proportion of samples currently taken in DHIA programs worldwide. It should be emphasized that the aim of this study was not to validate the instrument and its accuracy for official measurement purposes. However, the results of this preliminary assessment, in our opinion, were satisfactory and accurate enough allowing to perform the field study as hypothesized, and to apply DSCC in routine practice in cows with ≤50,000 cells/mL.

### 4.2. Field Trial—Data Description

The observed pattern for total and differential milk cell content showing an increase in DSCC as SCC increases was expected [10,16]. These data confirm that primiparous cows and fresh cows are, generally, the cows with the lowest SCC values in the herd [19,20]. The high frequency of cows with ≤50,000 cells/mL among animals in first lactation and in the first 60 DIM support the importance of having tools enabling an early identification of IMI in apparently healthy cows. Indeed, previous studies have already shown that, even at these low SCC levels, cows can still have an IMI [12,21]. The large difference in the correlation between cows with ≤50,000 cells/mL and >50,000 cells/mL suggests that, despite the biological relationship between these two traits, DSCC values give information that is not fully overlapping with the ones supplied by the SCC. The higher correlation observed in samples with >50,000 cells/mL can be easily explained by the presence of an inflammatory process and is characterized by an increase in the PMN proportion, as confirmed by our data (Table 2) and as suggested by [10,16]. The low correlation between the SCS and DSCC in very low SCC samples suggests that other biological mechanisms may regulate leukocyte proportions in healthy quarters.

### 4.3. Intramammary Infections and DSCC

The molecular analysis of the milk samples confirmed that about 20% of them harbored a pathogen, despite the very low SCC, including contagious ones, which was not unexpected [21,22]. These data emphasize the importance of microbiological analysis (conventional or molecular) also in milk samples with a low SCC, particularly in herds with contagious pathogens problems [22]. The results of these analyses show that DSCC did not increase our capability to identify IMI when the SCC is very low in individual cow milk. This outcome may be explained both from a methodological and immunological point of view. Indeed, DSCC as well as molecular analyses were performed in individual milk samples. Therefore, a dilution effect is probable. Indeed, the very low SCC suggests that any inflammatory process should be at the very beginning and, very likely, is confined in the infected quarter [23]. It should be also noticed that, despite DSCC values reporting the sum of the proportion of PMN and LYM, it has been shown that these latter ones are in the range of 1–19%, independently of the SCC of the samples [16]. Therefore, we expect a moderate increase mainly of PMN in infected quarters, and, once mingled with the milk of healthy quarters, it would be buffered by the very low PMN concentration in the milk of these other quarters. To confirm this hypothesis, it is necessary to perform studies on quarter milk samples, studies that are currently ongoing in our lab.

### 4.4. DSCC, Milk Composition and Yield

The analysis of data on milk composition showed that the herd had a relatively small influence on the milk components, while it was higher for milk yield. Our data confirm that even at low SCC levels an increase in SCC is associated with an increase in PMN, as expected [7,15], while IMI did not show any effects, confirming previous analysis regarding the association between DSCC and IMI.

These data suggest that DSCC could be a useful marker in very low SCC cows for estimating the milk secretion process. Indeed, it was possible to identify three clusters where DSCC means are very different among them and they are also characterized by significant changes in milk components. The relationship between changes in milk composition and increasing the SCC and PMN content of milk has been already shown [24,25]. However, these studies involved milk samples with a relatively high SCC and, in any case, higher than 50,000 cells/mL. It is also well-known that the presence of an IMI or a subclinical mastitis is associated with the increase in cellular content of milk and changes in milk composition [26,27]. It has been also suggested that the milk composition changes when the SCC is ≤50,000 cells/mL [12]. At the best of our knowledge, this is the first study that focused only on milk samples with a very low SCC and showing that changes in leukocyte proportions are associated with changes in milk composition under field conditions. The changes observed were statistically independent from the presence of an IMI, since the distribution of IMI were uniform along the range of DSCCs observed (12.2%–91.6%), and there was not any statistical association between the IMI and DSCC. 

Therefore, the changes in DSCC suggest that secretory tissues were exposed to the initial phase of an inflammatory process leading to the diapedesis of PMN in milk. The identification of the mechanisms behind these changes are out of the scope of this paper; however, it can be hypothesized that the changes could be related to any or a combination of the following mechanisms: a reduced blood–milk barrier [23]; the effects of serotonin concentrations, which are associated with an increased transfer of the paracellularly transported serum albumin, lactate dehydrogenase and SCC [28]; and/or the changes in the systemic and local factors that regulate tight junctions [29].

## 5. Conclusions

This study confirms that cows with a very low SCC (≤50,000 cells/mL) may harbor IMI; however, the DSCC did not increase our capability to identify them. Despite the low level of the SCC, milk fat, protein and casein significantly declined as the DSCC was raised. Therefore, a DSCC in low SCC cows may be suggested as a marker to identify early changes in milk composition, as a result of an alteration in the milk secretion mechanism.

## Figures and Tables

**Table 1:** Results of repeatability trials for DSCC ^1^ and SCC ^2^ in milk samples with ≤50,000 cells/mL.

Statistics	Trial 1		Trial 2		Trial 3		Trial 4		Trial 5	
	Instrument 1	Instrument 2	Instrument 1	Instrument 2	Instrument 1	Instrument 2	Instrument 1	Instrument 2	Instrument 1	Instrument 2
SCC										
Mean	40	39	43	44	46	48	41	43	44	44
Std. Dev.	3.62	2.37	2.09	2.97	2.54	3.82	2.09	2.67	2.48	2.78
CV % ^3^	9	6	5	7	6	8	5	6	6	6
Min	33	39	39	38	41	42	38	37	38	39
Max	47	48	47	51	50	53	45	47	48	49
Range	14	9	8	13	9	11	7	10	10	10
DSCC										
Mean	40.0	37.7	66.7	66.4	60.2	59.0	59.6	60.2	57.8	56.3
Std. Dev.	2.80	2.39	2.65	2.56	2.90	2.90	2.91	3.25	3.28	1.94
CV %	9	6	4	4	5	5	5	5	6	3
Min	35.3	34.7	61.1	61.4	54.1	54.7	54.2	54.7	52.1	52.5
Max	44.3	42.5	71.3	72.1	65.9	65.8	65.9	66.3	62.1	61.3
Range	9.0	7.8	10.2	10.7	11.8	11.3	11.7	11.6	10	8.8

^1^ DSCC: differential somatic cell count (%); ^2^ SCC: somatic cell count—values reported must be multiplied by a factor 1,000; ^3^ CV%: coefficient of variation.

**Table 2:** Descriptive statistics of total and differential somatic cell counts classified by SCC (threshold ≤50,000/>50,000 cells/mL).

Statistics	Samples ≤ 50,000 Cells/mL				Samples > 50,000 Cells/mL			
	SCC ^1^/mL	SCS ^2^ (Units)	DSCC ^3^ (%)	Yield (kg/d)	SCC/mL	SCS (Units)	DSCC (%)	Yield (kg/d)
Mean	28,600	4.41	50.89	35.25	506,070	5.34	67.97	30.04
Std. Dev.	11,418	0.20	13.38	9.24	1,100,636	0.49	14.24	9.81
Min	5,000	3.70	12.2	15.0	51,000	4.71	5.10	4.0
Max	50,000	4.70	91.6	72.0	18,242,000	7.26	93.9	69.0

^1^ SCC: somatic cell count; ^2^ SCS: somatic cell score (Log_10_ SCC/mL); ^3^ DSCC: differential somatic cell count.

**Table 3:** Distribution of samples by number of parturitions and SCC level (threshold ≤50,000/>50,000 cells/mL).

Cells/mL		Parity					Total
		1	2	3	4	>4	
≤50,000	N	460 ^a, 1^	266 ^b^	103^c^	41 ^c^	31 ^c^	901
	%	38.8%	30.2%	21.4%	14.4%	16.6%	29.8%
>50,000	N	726 ^a^	616 ^b^	379 ^c^	244 ^c^	156 ^c^	2,121
	%	61.2%	69.8%	78.6%	85.6%	83.4%	70.2%
Total	N	1,186	882	482	285	187	3,022
	%	39.2%	29.2%	15.9%	9.4%	6.2%	100.0%

^1^ Columns with a different superscript are statistically different following a χ^2^ test (*p* < 0.05), suggesting a statistical difference comparing the frequency of cows among table cells.

**Table 4:** Distribution of samples by days in milk and SCC level (threshold ≤50,000/>50,000 cells/mL).

Cells/mL		Days in Milk					Total
		5–60	61–120	121–180	181–240	>240	
≤50,000	N	238 ^a, 1^	227 ^a^	145 ^b^	122 ^b^	169 ^c^	901
	%	43.3%	41.3%	31.8%	28.4%	16.3%	28.8%
>50,000	N	312 ^a^	323 ^a^	311 ^b^	307 ^b^	868 ^c^	2,121
	%	56.7%	58.7%	68.2%	71.6%	83.7%	70.2%
Total	N	550	550	456	429	1037	3,022
	%	18.4%	18.4%	15.2%	14.3%	33.8%	100.0%

^1^ Columns with a different superscript are statistically different following a χ^2^ test (*p* < 0.05), suggesting a statistical difference comparing the frequency of cows among table cells.

**Table 5:** Distribution of the microbiological results within the DSCC quartiles.

Microbiological Results	DSCC ^1^ (%)			
	12.2–41.2	41.3–51.7	51.8–60.6	60.7–91.6
Negative	24.5%	24.4%	25.4%	25.8%
Major pathogens	27.8%	27.3%	25.1%	19.8%
Coagulase negative staphylococci	27.3%	30.0%	24.5%	18.2%

^1^ DSCC: differential somatic cell count.

**Table 6:** Analysis of variance results and least square means ± standard error of the mean calculated by a general linear model for milk yield, SCS and milk components.

**R^2^ Model (%)** **P of the Model**		**N**	**Milk Parameters**					
			**Yield (kg/d)**	**SCS ^2^ (units)**	**Fat %**	**Protein %**	**Casein %**	**Lactose %**
			55.1	15.4	26.5	45.3	46.3	25.8
			<0.0001	<0.0001	<0.0001	<0.0001	<0.0001	<0.0001
Herd contribution to R^2^ (%)			22.9	6.0	14.6	5.3	5.3	13.7
DSCC ^3^ (%)	P		0.2965	<0.0001 ^1^	0.0003	<0.0001	<0.0001	0.2113
Classes	12.2–41.2	227	34.5 ± 0.63	4.38 ± 0.019 ^a^	4.25 ± 0.074 ^a^	3.42 ± 0.027 ^a^	2.68 ± 0.024 ^a^	4.83 ± 0.015
	41.3–51.7	225	35.0 ± 0.63	4.44 ± 0.019 ^b^	4.11 ± 0.073 ^b^	3.39 ± 0.027 ^a^	2.65 ± 0.024 ^a^	4.84 ± 0.015
	51.8–60.6	228	36.7 ± 0.64	4.45 ± 0.019 ^b^	3.99 ± 0.075 ^b, c^	3.31 ± 0.027 ^b^	2.59 ± 0.025 ^b^	4.86 ± 0.015
	60.7–91.6	221	35.4 ± 0.69	4.49 ± 0.020 ^c^	3.96 ± 0.080 ^c^	3.30 ± 0.029 ^b^	2.58 ± 0.026 ^b^	4.86 ± 0.016
Parity (n)	P		<0.0001	0.1497	0.2902	0.0003	0.0003	<0.0001
Classes	1	460	29.5 ± 0.48 ^a^	4.42 ± 0.014	4.00 ± 0.056	3.40 ± 0.021 ^a^	2.67±0.018 ^a^	4.93±0.011 ^a^
	2	266	36.1 ± 0.57 ^b^	4.45 ± 0.017	4.10 ± 0.066	3.45 ± 0.024 ^b^	2.70 ± 0.021 ^a^	4.85 ± 0.014 ^b^
	3	103	36.0 ± 0.74 ^b^	4.46 ± 0.022	4.00 ± 0.085	3.39 ± 0.0.3 ^a, b^	2.65 ± 0.028 ^a^	4.83 ± 0.018 ^b^
	4	41	38.4 ± 1.07 ^c^	4.46 ± 0.032	4.04 ± 0.124	3.38 ± 0.046 ^c^	2.56 ± 0.041 ^b^	4.81 ± 0.026 ^b^
	>4	31	35.6 ± 1.24 ^b, c^	4.43 ± 0.036	4.24 ± 0.143	3.26 ± 0.053 ^c^	2.55 ± 0.047 ^b^	4.82 ± 0.030 ^b^
Days in milk (d)	P		<0.0001	<0.0001	<0.0001	<0.0001	<0.0001	<0.0001
Classes	5–60	248	37.6 ± 0.60 ^a^	4.37 ± 0.018 ^a^	3.98 ± 0.070 ^a^	3.31 ± 0.026 ^a^	2.41 ± 0.023 ^a^	4.87 ± 0.015 ^a^
	61–120	227	38.4 ± 0.63 ^a^	4.41 ± 0.018 ^b^	3.81 ± 0.073 ^b^	3.17 ± 0.027 ^a^	2.46 ± 0.024 ^b^	4.89 ± 0.015 ^a^
	121–180	145	35.7 ± 0.72 ^b^	4.44 ± 0.021 ^b, c^	3.93 ± 0.084 ^b^	3.32 ± 0.031 ^b^	2.59 ± 0.028 ^c^	4.86 ± 0.017 ^a^
	181–240	122	34.1 ± 0.78 ^c^	4.47 ± 0.023 ^c^	4.26 ± 0.091 ^c^	3.51 ± 0.034 ^c^	2.77 ± 0.029 ^d^	4.82 ± 0.019 ^b^
	>240	169	29.8 ± 0.70 ^d^	4.52 ± 0.021 ^d^	4.40 ± 0.081 ^c^	3.66 ± 0.030 ^d^	2.88 ± 0.027 ^e^	4.80 ± 0.017 ^b^
IMI ^4^	P		<0.0978	0.3555	0.1686	0.8360	0.8634	0.7921
Classes	Negative	714	35.1 ± 1.04	4.44 ± 0.012	4.10 ± 0.051	3.37 ± 0.018	2.61 ± 0.016	4.85 ± 0.010
	CNS	99	36.3 ± 0.73	4.45 ± 0.031	3.93 ± 0.121	3.34 ± 0.045	2.63 ± 0.040	4.85 ± 0.017
	Major path.	88	33.9 ± 0.73	4.44 ± 0.022	4.19 ± 0.085	3.36 ± 0.031	2.63 ± 0.028	4.84 ± 0.017

^1^ Row means with a different superscript are statistically different (*p* < 0.05), suggesting a statistical difference comparing milk component means among the classes of each factor; ^2^ SCS: somatic cell score (Log_10_ SCC/mL); ^3^ DSCC: differential somatic cell count; ^4^ IMI: intramammary infection.

**Table 7:** Comparison of milk component means among the three clusters defined by agglomerative hierarchical clustering.

Cluster	N	DSCC ^2^ (%)		SCS ^3^ (unit)		Fat (%)		Protein (%)		Casein (%)		Lactose (%)	
		Mean	Std. Dev.	Mean	Std. Dev.	Mean	Std. Dev.	Mean	Std. Dev.	Mean	Std. Dev.	Mean	Std. Dev.
A	274	35.04 ^a. 1^	6.14	4.36 ^a^	0.20	4.19 ^a^	0.82	3.46 ^a^	0.37	2.71 ^a^	0.33	4.86 ^a^	0.17
B	518	54.73 ^b^	6.32	4.43 ^b^	0.19	4.02 ^b^	0.86	3.36 ^b^	0.35	2.63 ^b^	0.31	4.90 ^b^	0.17
C	109	72.47 ^c^	5.38	4.60 ^b^	0.20	3.99 ^b^	0.72	3.27 ^c^	0.35	2.55 ^c^	0.30	4.90 ^b^	0.19

^1^ Row means with a different superscript are statistically different following an ANOVA LSD test for comparison of milk component means (*p* < 0.05), suggesting a statistical difference when comparing the clusters. ^2^ SCS: somatic cell score (Log_10_ SCC/mL) ^3^ DSCC: differential somatic cell count.
